# NAO TAKES A BOW: USING SOCIAL ROBOTS TO ENHANCE THE MOOD OF OLDER ADULTS LIVING IN RESIDENTIAL CARE SETTINGS

**DOI:** 10.1093/geroni/igz038.561

**Published:** 2019-11-08

**Authors:** Noelle L Fields, Julienne Greer, Ling Xu

**Affiliations:** University of Texas at Arlington, Arlington, Texas, United States

## Abstract

Social robots have been utilized in research with older adults, however, few studies have integrated participatory arts (e.g. theatre) into social robotic platforms with this population. An interdisciplinary team designed an intervention integrating theater and social robotics with the aim of improving the mood of older adults. A purposive sample of persons age 65 and older (N = 15) participated in this 3-session pilot study that involved a Shakespeare activity using the robot, NAO. Mixed methods included interview questions as well as short survey measures of depression, loneliness, and a simplified face scale for mood pre and post each session. Results from Repeated Measurement Analysis of Variance (ANOVA) showed that face scale scores significantly decreased across six time periods (F = 2.72 (5, 50), p <. 05) and this decrease marginally differed between participants with dementia (M = 2.50, SD = 1.73) and those without dementia (M = 1.62, SD = 0.52). In addition, depression scores marginally significantly decreased after intervention (F = 2.28 (5, 40), p <. 10) and these declines were also marginally significantly different for participants with (M = 0.67, SD = 0.58) or without dementia (M = 0.86, SD = 0.69). Qualitative findings suggest that participants were highly engaged and responsive to the intervention. We discuss the promising aspects of using social robotics as a platform for participatory arts interventions with older adults and offer recommendations for future interdisciplinary studies involving the use of innovative technology in residential care settings.

